# Retraction of: LINC00514 drives osteosarcoma progression through sponging microRNA-708 and consequently increases URGCP expression

**DOI:** 10.18632/aging.206066

**Published:** 2024-08-15

**Authors:** Dapeng Yu, Xiangyan Xu, Sufen Li, Kai Zhang

**Affiliations:** 1 Department of Spine Surgery, Shandong Provincial ENT Hospital, Shandong Provincial ENT Hospital Affiliated to Shandong University, Ji’nan 250022, Shandong, China; 2 Department of Traumatic Orthopedics, Shandong Provincial ENT Hospital, Shandong Provincial ENT Hospital Affiliated to Shandong University, Ji’nan 250022, Shandong, China; 3 Orthopedic and Soft Tissue Surgery, Shandong Cancer Hospital and Institute, Shandong First Medical University and Shandong Academy of Medical Sciences, Ji’nan 250117, Shandong, China; 4 Department of Orthopedics, Shandong Provincial Third Hospital, Ji’nan 250031, Shandong, China

**Keywords:** long noncoding RNA, osteosarcoma, microRNA

**This article has been retracted:** Aging has completed its investigation of this paper. Several concerns were raised about this paper, including overlap with unrelated papers from different institutions. We found that two panels in **Figure 7G** “The migratory and invasive abilities of the aforementioned cells were assessed by transwell migration and invasion assays” are duplications of Figure 1H published earlier in an unrelated paper [1] and Figure 6C published in a second unrelated paper [2]. In addition, **Figure 8A** “Representative image of subcutaneous tumor xenografts collected from the sh-LINC00514 and sh-NC groups” contains the same distinctively scratched ruler that was used to measure xenograft tumors in three other papers from unrelated teams of authors [3–5], which have already been retracted. Moreover, we discovered that the formats of two of those papers [4, 5] and the discussed paper are nearly identical, with some figures being almost indistinguishable. The Scientific Integrity office at Aging contacted the authors, but the authors did not respond to requests to clarify, and the paper was retracted based on an Editorial decision. The Scientific Integrity office also notified the authors’ Institutions about this retraction and added their names to an Editorial Warning list.
